# Development and validation of a novel cosmetics safety assessment scale (CSAS): Factual understanding of cosmetic safety and fostering international awareness

**DOI:** 10.1371/journal.pone.0276938

**Published:** 2022-11-10

**Authors:** Ammar Abdulrahman Jairoun, Sabaa Saleh Al-Hemyari, Moyad Shahwan, Shazia Jamshed, Justyna Bisgwa

**Affiliations:** 1 School of Pharmaceutical Sciences, Universiti Sains Malaysia, Pulau Pinang, Gelugor, Malaysia; 2 Health and Safety Department, Dubai Municipality, Dubai, UAE; 3 Pharmacy Department, Emirates Health Services, Dubai, United Arab Emirates; 4 Department of Clinical Sciences, College of Pharmacy and Health Sciences, Ajman University, Ajman, United Arab Emirates; 5 Center of Medical and Bio-allied Health Sciences Research, Ajman University, Ajman, United Arab Emirates; 6 Department of Clinical Pharmacy and Practice, Faculty of Pharmacy, Universiti Sultan Zainal Abidin, Kuala Terengganu, Malaysia; 7 Justyna Bisgwa is an Independent Cosmetics Safety and Regulatory Affairs Specialist, Opole, Poland; Shandong University of Science and Technology, CHINA

## Abstract

**Background:**

Falsified cosmetics are increasingly common especially online through social media networks and mobile applications.

**Objectives:**

This study developed and validated a novel tool to evaluate the safety of cosmetics and personal care products in the United Araba Emirates (UAE).

**Method:**

This is methodological validation study and the data were derived from a cross-sectional study conducted on students and staff at Ajman University (AU) in the UAE. The study sample was selected via simple random sampling. The link to the survey was sent to potential respondents via email, and the responses were analysed using SPSS version 26. Content validity, factor analysis, and known group validity were employed to construct and validate an instrument that will enable the identification of cosmetics safety. The instrument’s reliability was evaluated using test-retest reliability, internal consistency, item internal consistency (IIC), and intraclass correlation coefficients (ICCs).

**Results:**

The study sample included 978 participants. The content validity index for the final 24-item scale was 0.84. The Kaiser-Meyer-Olkin value was 0.959 with a statistically significant Bartlett’s test of sphericity (p <0.001). Factor analysis presented a three-component model. PCFA analysis found good fit values with 0.960 for the normed fit index, 0.977 for the comparative fit index, and 0.987 for the Tucker Lewis Index. All values were in excess of 0.95, and the root mean square error of approximation was below 0.06 (0.03); thus, the model had a good fit. Cronbach’s alpha also showed good consistency of the overall instrument (0.963), and all factors had a Cronbach’s alpha above 0.70. Each item on the instrument met the IIC correlation standard of ≥ 0.40, and there were good overall ICC statistics of 0.963 (0.959–0.966) for the instrument as a whole with statistical significance (p < 0.001). The instrument’s test-retest reliability was assessed by correlating the respondents’ identification scores at two time points with a four-week gap revealing a correlation coefficient of 0.870 (p-value <0.01). Participants holding a bachelor’s degree were more likely to be able to identify safe and authentic cosmetics than those with a high-school educational level (p = 0.015).

**Conclusions:**

This study developed a novel validated instrument to determine the safety of cosmetics. The final questionnaire uses 24 items on three dimensions (13 items on hazard information, eight items on product identity, and three items on product handling and storage). The tool is concise and easy to complete, and it is suitable for use among the general population. The use of this instrument can promote greater collaboration between the consumer health regulatory authorities and inspection authorities thus increasing consumer satisfaction and public participation.

## Introduction

The growing global cosmetics and personal care product market is currently valued at $532 billion **[1]** and motivates research and development of new cosmetic products. In the European Union, the sale of these products is governed by EC Regulation No 1223/2009 established by the European Parliament and Council on 30 November 2009. The regulation considers cosmetics and personal care products to be any product, mixture or substance that comes into contact with any external human body part, e.g., skin, hair, lips, nails, external genitalia, or the teeth or oral cavity’s mucous membranes for the purpose of cleaning, maintenance, protection, or altering their appearance or odor.” Based on this, any cosmetic product for sale on the EU market must be safe for human consumption under common and reasonably predictable conditions and based on the labelling, presentation, usage and disposal instructions, warnings, and any other information **[2]**.

Similarly, the regulatory authorities of the UAE require every product intended for human consumption, including cosmetics, to be registered with the respective emirates’ municipality and be in compliance with the regulations. The UAE, including Dubai, has banned the manufacture, import/export, and sale of unregistered cosmetics and personal care products. The product registration process enables the regulatory authorities to gather information on the products to assess their safety in the interest of consumers **[3]**. Cosmetic products are categorized according to their use including rinse-off or leave-on products. These groups, in turn, contain numerous subcategories such as skin products (e.g., washing gel, cream, mask, scrub, and serum), hair products (e.g., shampoo, conditioner, and hair dye), lip products (e.g., lipstick, lip balm), nail products (e.g., lacquer, conditioner), and oral cavity products (e.g., toothpaste and mouthwash).

While all ingredients in cosmetic products must meet the regulatory requirements **[2]**, many substances are only allowed at a certain limit because of dose-dependent toxicity. Additional important aspects include the potential for long-term **[4,5]** and acute adverse side effects such as contact dermatitis and allergic reactions **[6].** Moreover, consumers’ daily continuous exposure to a broad range of cosmetics as well as diverse chemicals from different sources raises concerns about the “cocktail effect” referring to potential synergistic interactions between various substances. Furthermore, the “additive effect” similarly comes into play as certain ingredients may be present in several different products that consumers use on a daily basis **[7,8].** Compounding these issues, falsified or counterfeit cosmetics have become increasingly prevalent on the market due to the proliferation of sales outlets including online through mobile applications and social media platforms. Falsified cosmetics can refer to adulterations in the product composition, false information on the label, or both.

The cosmetics market in the United Arab Emirates (UAE) is expected to expand to USD 3 billion by 2025, in part because of the country’s increasingly youthful demographic. In 2019, Dubai consumed the most cosmetics products in the UAE, primarily due to its high quality of life and high gross domestic product (GDP). Meanwhile, the UAE’s next-largest market, Abu Dhabi, with a similarly youthful and growing population and a strong economy, also has a strong cosmetics market, with numerous international brands recently being introduced. The Covid-19 pandemic in 2020 caused an unprecedented surge in e-commerce as people remained home to avoid the virus, and cosmetics products were among the most sought-after purchases **[9].**

The use of falsified products that contain banned ingredients or substances at concentrations above permitted levels can lead to significant side effects and adverse health issues. For example, a study on cosmetics and personal care products in the UAE determined that 13% (n = 9) of the tested products contained formaldehyde beyond the recommended level, but none of these products was labelled as containing free formaldehyde or formaldehyde releasers **[10].** Falsified cosmetics manufactured or stored under unsafe conditions can also become contaminated by pathogens, e.g., bacteria or mould. Research on the UAE cosmetics and personal care product market found that 5% of the 100 tested products were contaminated by aerobic mesophilic bacteria while 13% contained yeast or mould **[11].**

Product labels bearing false information, inadequately giving the manufacturer’s name or address, or not listing all the ingredients and their quantities are further issues related to falsified cosmetics. Another study on the safety of cosmetics on the market in Dubai found that 6 out of the 102 tested alcohol-based hand sanitizers contained undeclared or unlisted methanol while other samples contained less than 60% alcohol despite being labelled as having a 70% alcohol content **[12–14].** Similar research in Ajman, UAE led to the closure of two manufacturers following the seizure of substantial quantities of fake medical sterilizer on the premises. The product, though labelled a medical sterilizer, was in fact a body perfume spray **[15].**

Another study in the UAE context evaluated 125 cosmetics and personal care products. Cetrimonium chloride was found to be above the recommended level for rinse-off hair products in five (4%) products, above the recommended level for leave-on hair products in 10 (8%) products, and above the recommended level for use as a preservative in cosmetics products in 24 (61.5%) products **[16].**

Additional evaluations found that 10 of the tested 50 cosmetics products had total fluoride levels exceeded the recommended concentration of 0.15%, and 12 products had a total fluoride concentration below the levels claimed by their labels. Furthermore, 22 of the products tested contained less than 1000 ppm fluoride **[17].** Meanwhile, a study in the UAE indicated that the manufacturers of cosmetics products that use cannabinoids should produce batch quality certificates to ensure that consumers are sufficiently informed about the tetrahydrocannabinol levels of their purchased products **[18].**

In light of the above, consumers require a new way to determine the quality of the cosmetics products they purchase, including the ability to detect fake cosmetics in line with the labelling regulations of various jurisdictions, such as the US FDA, European Union, and Dubai Municipality Health and Safety Department **[19–22].**

Several initiatives from various countries, including in Africa, were used to address the falsification of medicines **[23,24].** These included approaches such as amplicon metabarcoding (AMB) and DNA barcodes, both of which allow falsified products to be identified during routine monitoring **[25–28].** With methods such as AMB not sufficiently mature for application, this study gathers and assesses the cosmetic product labelling regulations from a variety of health regulatory authorities, specifically those of the US, the EU and the UAE. The aim hereby is to develop a validated self-reporting tool that can facilitate the detection of falsified cosmetics products during routine inspections for both the UAE population and consumers in other countries. Utilized in conjunction with effective training and education, such a tool could contribute significantly to enhancing consumer safety in terms of the correct usage of such products. We expect that the authorities in other countries will benefit from our findings in addressing the growing issue of fake cosmetics products.

## Materials and methods

### Study design and setting

**Fig 1:**
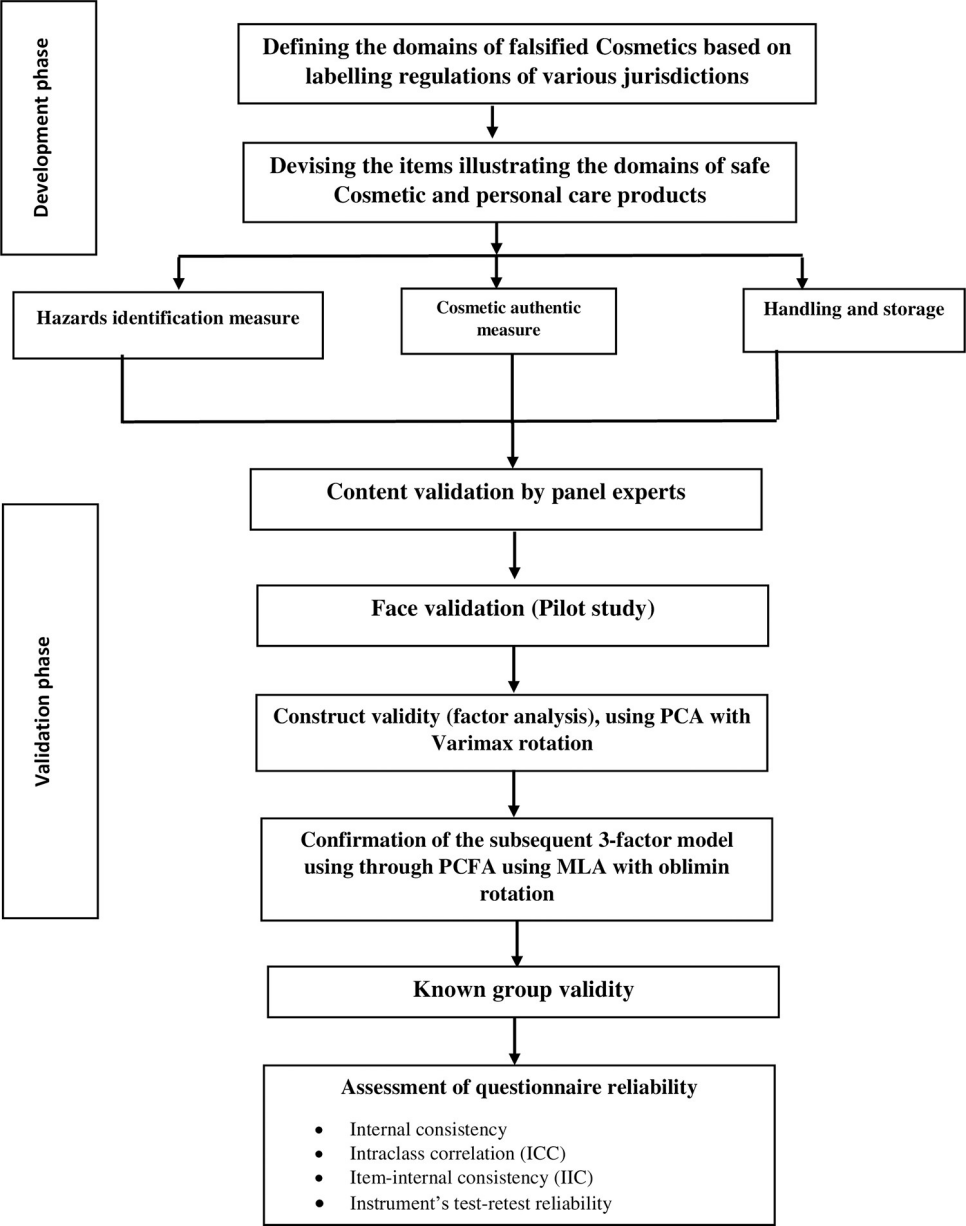
Flow chart for development and validation process.

This is methodological validation study and the data were derived from a cross-sectional study conducted on students and staff at Ajman University (AU) in the UAE. Its aim was to develop and validate a novel self-reporting English-language instrument that can measure the UAE public’s ability to identify the cosmetic safety. Our methodological approach was similar to that in our recent work on dietary supplements **[29].** The questionnaire was in the English language because of its role as the international language of science as well as of scientists around the world. By using English, our developed instrument should be able to offer the widest possible benefit. The development and validation of the tool are illustrated in **Fig 1**. An online survey questionnaire was used; the respondents were sent a link to the questionnaire by email. The data were collected from January 20^th^ 2021 to May 10^th^ 2021.

### Study participants (inclusion and exclusion criteria)

The target population was both UAE nationals and non-national residents of the UAE. All potential respondents aged 18 years and older and willing to participate were included. Those below the age of 18 years or who did not indicate a willingness to participate were excluded.

### Pilot testing

A pilot study was conducted at Ajman University from December 25^th^ 2020 to January 12^th^ 2021. Of the 300 potential pilot study respondents, 250 completed the questionnaire (response rate = 83.3%), and no problems were reported. The pilot study results were used to estimate the necessary sample size as well as to assess the test-retest reliability.

### Sample size and sampling technique

Sample size calculation was based on the question, “Do you know how to identify the cosmetic product safety?”. Around 66% of the respondents in the pilot study answered this question affirmatively. The alpha level was set to 5% to obtain a 95% confidence interval (CI), and the precision (D) of the 95% CI was set to 5% to obtain a maximum 10% width of the 95% CI. Based on this—and assuming a non-response rate of around 70%—a sample size of n = 1150 participants was required. The Ajman University Admission and Registration Department provided an Excel spreadsheet containing specific information of all staff and students including name, college, study year, and email address. Potential participants were selected from this list using simple random-sample selection based on their ID numbers and stratified by college and department.

### Questionnaire administration

The preselected respondents were drawn from the admission, and the questionnaire was sent to their emails after registration. The first page of the survey outlined the study’s nature and purpose; participants who clicked to continue to the following page were assumed to have given their consent to participate. Participants who did not initially respond to the invitation emails were sent reminder emails every month during the survey period. Upon completion of the survey, the respondents received a “Thank You” message. Participants received no incentives for completing the survey.

### Ethical considerations

The study received approval from the Ajman University Institutional Ethical Review Committee (P-H-S-2021-2-10). Participation was voluntary, and the participants were informed of the study purpose on the first page of the survey. An electronic link to the questionnaire was added to the cover letter. The participants first choose to accept or decline their participation by responding to a question before responding to the items in the questionnaire. Those that agreed to participate signed a written statement of informed consent. The respondents’ identities were not recorded, and they were assured of confidentiality.

### Research instrument development and conceptualization

#### Face and content validity

The scale used in the first questionnaire draft was assessed for both face and content validity by an expert panel comprising four ISO 22716 Auditors, two cosmetics safety and regulatory affairs specialists, and two academics. Public opinion on the questionnaire was also sought. Each expert on the panel was asked to assess whether each questionnaire item was essential; this produced the content validity index (CVI) and content validity ratio (CVR). A CVR of 0.78 or above is considered to represent good content validity. Any item not reaching this threshold was eliminated from the final instrument. The mean CVR values for all items that met this threshold were retained and were then calculated to achieve the CVI **[30,31].**

#### Construct validity

The questionnaire’s construct was assessed using exploratory factor analysis (EFA) as conducted via principal component analysis (PCA) with Varimax rotation. The Kaiser-Meyer-Olkin (KMO) test for sampling adequacy and Bartlett’s test of sphericity were used to determine the number of factors. Eigenvalues of 1 and items loaded with at least 0.40 with no cross-loading of items above 0.40 were considered to be the criteria for construct validity **[32].** Partial confirmatory factor analysis (PCFA) via maximum likelihood analysis (MLA) with oblimin rotation was used to confirm the model. The incremental fit indices (comparative fit index (CFI), normed fit index (NFI), and Tucker Lewis Index (TLI)) were calculated. Finally, the absolute fit index was calculated via the root mean square error of approximation (RMSEA) **[33,34].**

#### Internal consistency and reliability analyses

The scale’s internal consistency was assessed using Cronbach’s alpha based on a criterion of at least 0.70 **[35].** Intraclass correlation coefficients (ICCs) were used to determine the test-retest reliability, utilizing interpretation criteria according to Rosner: ICC < 0.40 is poor agreement, 0.40 ≤ ICC < 0.75 is fair-good agreement, and ICC ≥ 0.75 is excellent agreement **[36].** Pearson’s correlation coefficient was used to measure the item internal consistency (IIC) referring to the relationship between the individual items and their respective domains or factors. Each item needed to demonstrate a correlation between r ≥0.4 and its adjusted scale score **[37].** Pearson’s correlation coefficient (ρ) was also used to assess the test-retest reliability following a four-week gap; a ρ above 0.75 and a p-value below 0.05 were showed a significant correlation **[33,34].**

#### Known group validation

We assumed that respondents with a higher education background were more likely to have the ability to recognize the cosmetics safety. A one-way ANOVA was used to assess the known group validity with a p-value less than 0.05 considered to be statistically significant.

#### Statistical analysis

The data were analysed using SPSS version 26. The respondents gave their responses to the items using a 5-point Likert scale (0 = “Never”, 1 = “Rarely”, 2 = “Sometimes”, 3 = “Often” and 4 = “Always”). The demographic and baseline characteristics of the sample were summarized using frequencies and percentages, and the difference in the identification rates of safe cosmetics was based on the level of education and was measured using one-way ANOVA. A p-value less than 0.05 was used to show statistical significance.

## Results

### Demographic details of the participants

Here, 978 respondents participated in the study and completed the questionnaire: 41.1% (n = 402) were male and 58.9% (n = 576) were female; 159 (21.5%) were aged 18 to 24, 210 (21.5%) were aged 25 to 34, 153 (15.6%) were aged 35 to 44, 276 (28.2%) were aged 45 to 54, 111 (11.3%) were aged 55 to 64 and 69 (7.1%) were aged 65 or older. In addition, 279 (28.5%) were high school education holders, and 699 (71.5%) had bachelor`s degrees. The nationality of participants included 15 (1.5%) were from Western Europe, 27 (2.8%) from South Asia, 693 (70.9%) from North America, 18 (1.8%) from North Africa, 27 (2.8%) from the Middle East, 66 (6.7%) from Europe, 12 (1.2%) from UAE, 30 (3.1%) from East Asia/Pacific, 9 (0.9%) from Central Asia, 3 (0.3%) from Australia, 33 (3.4%) from Africa, and 45 (4.6%) from other countries **(Table 1).**

**Table 1:** Demographic information (n = 978).

Demographics	Groups	Frequency	Percentage
**Gender**	Male	402	41.1%
	Female	576	58.9%
**Age groups**	18 to 24	159	16.3%
	25 to 34	210	21.5%
	35 to 44	153	15.6%
	45 to 54	276	28.2%
	55 to 64	111	11.3%
	65 or older	69	7.1%
**Nationality**	Western Europe	15	1.5%
	South Asia	27	2.8%
	North America	693	70.9%
	North Africa	18	1.8%
	Middle East	27	2.8%
	Europe	66	6.7%
	Emirati	12	1.2%
	East Asia/Pacific	30	3.1%
	Central Asia	9	0.9%
	Australia	3	0.3%
	Africa	33	3.4%
	Other	45	4.6%
**Educational level**	High school	279	28.5%
	Bachelor`s degree	699	71.5%

### Validation analysis

#### Face/Content validity

The first draft of the instrument comprised 46 items. After the draft was evaluated by the expert panel, seven of these items were modified based on their feedback. We subsequently determined that 22 items did not meet the minimum CVR of 0.78, and these were thus eliminated from the scale. The final 24-item scale had a CVI of 0.840.

#### Construct validity (factor analysis)

**Fig 2:**
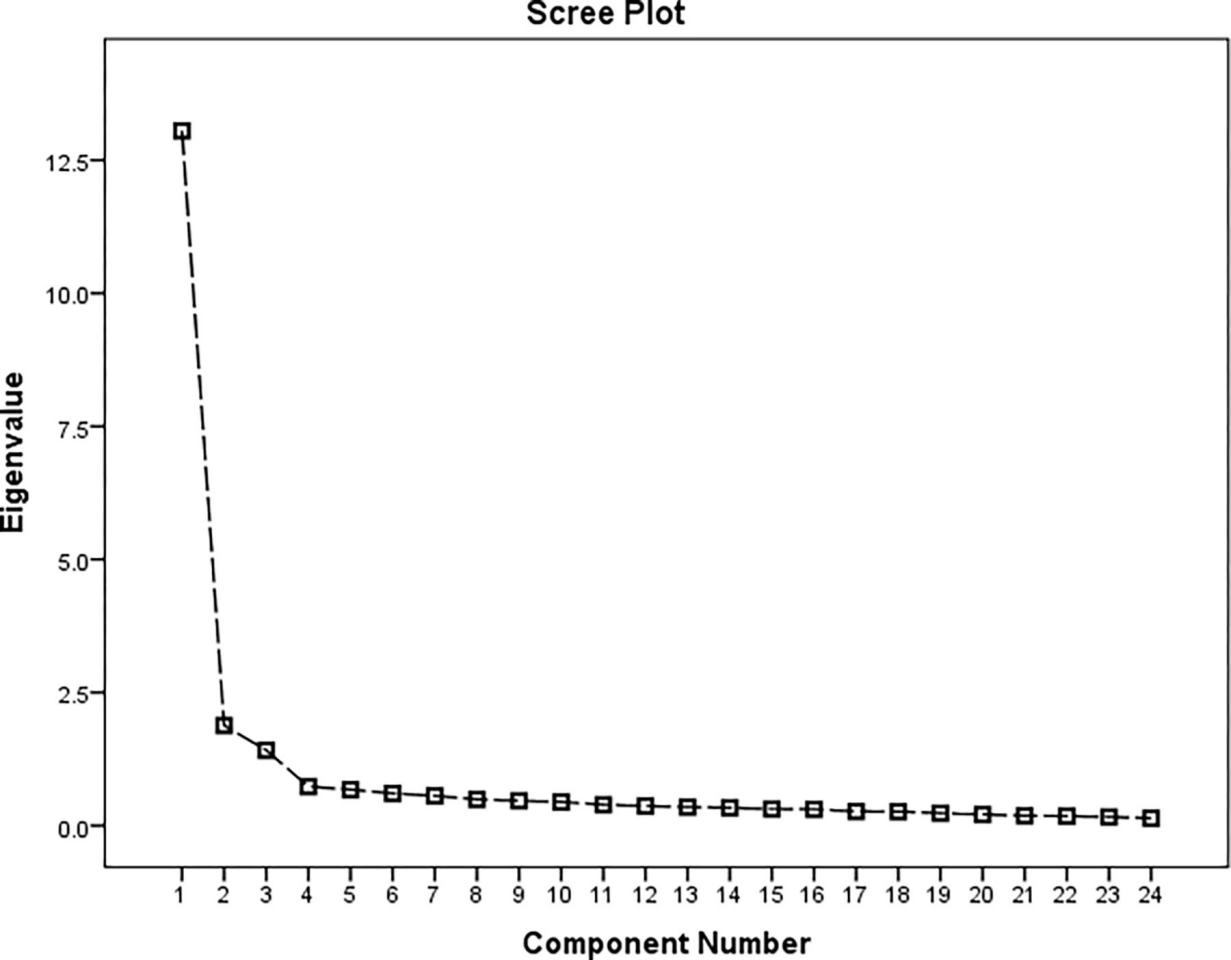
Scree plot and component factors resulting from the PCA.

EFA using PCA with Varimax rotation was used to assess the structure of the instrument’s factors. The KMO measure of sampling adequacy was 0.959, and Bartlett’s test of sphericity indicated significance (p-value< 0.001). A three-factor solution was determined to have eigenvalues above 1 and accounted for 68.1% of the variance with factor 1 presenting 54.4% and factors 2 and 3 constituting 7.8% and 5.9%, respectively. Items that factor loaded > 0.4 onto one component and demonstrated non-salient loading < 0.4 on another component were considered to be a single factor. A clear factor structure was hereby obtained **(Table 2 & Fig 2)**. Confirmation of the subsequent 3-factor model was achieved through PCFA using MLA with oblimin rotation. The KMO was 0.892, and Bartlett’s test of sphericity demonstrated significance (p-value<0.001). The distribution curve of the non-salient factor loading was normal with a mean value of 0.1. The null model χ2 was 8225.25, and the χ2 of the implied model was 944.67. The PCFA obtained values of 0.960 for NFI, 0.977 for CFI, and 0.987 for TLI; all values were greater than 0.95, and the RMSEA value was 0.03 (i.e., less than 0.06). Thus, the model had a good fit.

**Table 2:** Content validity ratio and factor structure emerging from principle factor analysis.

	Item content	CVR	Component			Communalities
			1	2	3	
**Factor 1** Hazards identification measure	Cautionary statement “Extremely flammable aerosol”	0.82	0.796	0.273	0.189	0.744
	Cautionary statement “Keep away from heat/sparks/open flames/hot surfaces”	0.85	0.795	0.361	0.065	0.766
	Information concerning particular hazards for accidental ingestion	0.88	0.793	0.303	0.210	0.765
	Information concerning particular hazards for accidental and prolonged inhalation	0.84	0.773	0.277	0.204	0.716
	Warning “Keep out of reach of children”	0.78	0.765	0.271	0.132	0.675
	Information concerning particular hazards for contact with skin/mucous membranes	0.82	0.763	0.167	0.347	0.730
	Information concerning particular hazards for contact with eyes	0.91	0.724	0.299	0.310	0.709
	Information on hazardous ingredients	0.79	0.717	0.323	0.242	0.677
	Cautionary statement “Do not pierce or burn, even after use”	0.81	0.706	0.411	0.122	0.683
	Information on conditions for safe storage	0.82	0.676	0.211	0.400	0.661
	Restrictions on use on use by specific groups of consumers	0.83	0.594	0.293	0.298	0.528
	List of ingredients or reference to enclosed or attached information about list of ingredients	0.79	0.569	0.341	0.395	0.595
	Relevant identified used, recommended use, and restrictions on use	0.92	0.521	0.284	0.351	0.564
**Factor 2** Cosmetic authentic measure	Batch number	0.81	0.211	0.873	-0.030	0.808
	Barcode	0.80	0.269	0.784	0.050	0.690
	Name and address of the manufacturer of the product	0.87	0.282	0.735	0.250	0.683
	Registration of the product in the country by the concerned regulatory authority	0.91	0.324	0.720	0.189	0.659
	Nominal content of the product	0.85	0.265	0.710	0.183	0.608
	Country of origin	0.93	0.321	0.667	0.282	0.691
	Cosmetic product claims and indications match with the active ingredients of the product	0.85	0.387	0.588	0.368	0.632
	Premises and shops selling the cosmetics assure that the products meet the proper specifications throughout its shelf life	0.88	0.356	0.568	0.382	0.595
**Factor 3** Handling measure	Cosmetic container is safely sealed	0.81	0.217	0.027	0.828	0.733
	Cosmetic container and closure protect the product from the outside environment	0.79	0.251	0.174	0.808	0.746
	Container and the closure are appropriate for the cosmetic product inside	0.81	0.269	0.251	0.664	0.689

#### Reliability analysis

Internal consistency (Cronbach’s alpha), IIC, and ICC were used to assess the reliability of the developed instrument. All factors demonstrated Cronbach’s alpha coefficients above 0.70 with 0.963 for the full instrument. Meanwhile, all items met the IIC correlation standard of ≥ 0.40. Finally, the instrument presented good ICC statistics of 0.963 (95% CI 0.959–0.966) as well as statistical significance (p < 0.001). The ICC for the factors ranged between 0.811 and 0.956. The reliability of factor 1 was reported at 0.956 with 95% confidence interval of (0.952–0.960). Factor 2 had an alpha value of 0.921; ICC = 0.913–0.928 for 95% CI. Factor 3 had an alpha value of 0.811, ICC = 0.789–0.831 for 95% CI; further details are given in Table 3. The instrument’s test-retest reliability was measured through a correlation between the participants’ identification scores for counterfeit and substandard cosmetics at time points 1 and 2 with a four-week gap; the correlation coefficient was 0.870 (p-value < 0.01).

**Table 3:** Reliability assessment criteria of the study scale (n = 1280).

Subscale	No. of items	Mean ± SD	Cronbach’s α	IIC	ICC (95% CI)
**Factor 1**	13	45.9 ±13.4	**0.956**	**0.728–0.865**	**0.956 (0.952–0.960)**
**Factor 2**	8	24.9 ± 8.5	**0.921**	**0.754–0.831**	**0.921 (0.913–0.928)**
**Factor 3**	3	11.8 ± 2.9	**0.811**	**0.841–0.866**	**0.811 (0.789–0.831)**
**Total**	24	82.7 ± 22.6	**0.963**	**---------------**	**0.963 (0.959–0.966)**

**Abbreviations:** SD, (standard deviation); IIC, item-internal consistency; ICC, intraclass correlation (consistency ICC from 2-way mixed model).

#### Known group validity

Known group validity was evaluated using a one-way ANOVA comparing the difference in the cosmetic safety identification scores of participants with different educational levels; see Table 4. This difference was found to be statistically significant with participants holding a bachelor’s degree being more likely to be able to identify counterfeit and substandard cosmetics than those with a high-school education (p = 0.015).

**Table 4:** Substandard and falsified cosmetic identification scores by education level.

Education level	Cosmetic safety identification scores.			
	Mean	± SD	95% confidence interval	
			Lower limit	Upper limit
High school	79.8	23.6	77.09	82.67
Bachelor`s degree	83.7	22.1	82.13	85.41

## Discussion

Tackling the issue of counterfeit and substandard cosmetics and personal care products remains a substantial challenge in public health. It is currently impossible to quantify the proliferation of these fraudulent products, and thus the critical first step towards overcoming this problem is creating an awareness of it at the local and global levels thus leading to the development of an effective solution. Here, we developed and validated a self-administered questionnaire-based instrument through which consumers and authorities can evaluate the safety of the cosmetics and personal care products on the market. To the best of our knowledge, our study is the first to outline the design and development stages of a validated instrument for this purpose that has good content validity, face validity, and reliability.

Cosmetics and personal care product manufacturers, suppliers, importers, wholesalers, distributors, and retailers must ensure that the labelling on their products complies with the information labelling standards set by the relevant regulatory authorities. In particular, all products must bear labels that list all ingredients including those that can cause an allergic reaction or have any other adverse effects. This labelling must follow a standard format to ensure the comparability of the various products on the market.

This work developed an instrument to assess the safety of cosmetics products based on the labelling requirements approach. Hereby, the conceptual model comprised three dimensions: the hazards identification measure, the product authentic measure, and the handling and storage measure. The respondents used a 5-point Likert scale to rate how frequently they examine the information provided by the labels of cosmetics and personal care label to check that all necessary details are present. In this context, any product not meeting the labelling requirements should be considered non-compliant with national health and safety requirements.

The validity of the instrument was addressed in several stages. Initially, to ensure that the content was appropriate, the items in the instrument were suggested and developed by a specialized committee drawn from several fields. Subsequently, the CVI was measured and shown to be valid (0.84). Finally, the instrument’s length and the time required for completion were measured and shown to contribute to the high response rate and the accuracy of the participants’ answers.

Factor analysis was used to examine whether it was possible to extract the underlying dimensions in support of the conceptual model. As conceptualized in the model, three distinct factors emerged each with eigenvalues greater than 1. This explained 68.1% of the total variance. The KMO value of 0.959 as well as the statistical significance of Bartlett’s test of sphericity (p < 0.001) further indicate that the correlation matrix was factorable. First, factor 1 assessed the respondents’ ability to identify potential hazards in cosmetics such as identified use, recommended use, use restrictions, information on specific hazards, and information on hazardous ingredients. Second, factor 2 measured their ability to identify the signs of authentic products such as manufacturing details, shelf life, barcode, batch number, country of origin, and claims and indications. Finally, factor 3 evaluated their ability to identify how to handle and store such products. This three-factor solution was confirmed via PCFA analysis and found to have a good fit; therefore, the purification of this measurement scale was more stringent.

The resulting instrument demonstrated good overall reliability by satisfying the criteria for the individually examined reliability issues. There was also a high rate of return for fully completed questionnaires highlighting the careful and willing responses of the participants. The Cronbach’s alpha for the overall instrument was good (0.963). The IIC for the items within each domain demonstrated relatively good Cronbach’s alphas for the three factors (0.811 to 0.956). The test-retest results further indicated the satisfactory temporal stability of the instrument. Overall, the instrument showed stable reliability.

The instrument also demonstrated the ability to distinguish between groups with different educational levels highlighting statistically significant differences in terms of their scores. Specifically, participants with a bachelor’s degree returned significantly higher scores than those with a high-school education suggesting that they could distinguish safe cosmetics and personal care products.

The instrument developed here could be further utilized for follow-up studies. Such research could produce a more detailed map of the channels through which falsified cosmetics are traded as well as facilitate the analysis of their impacts on consumers, the industry, and government. This analysis revealed the true effects of falsified and substandard cosmetics and personal care products thus offering guidance on how to further develop and strengthen current risk-based enforcement strategies. An additional topic of concern in this field is that the trade in falsified products has proliferated due to the use of free trade zones that offer a space for fake products to be repackaged to mislead authorities and consumers regarding their true origin. Similarly, the use of online shopping channels with their global reach and rogue online pharmacies has facilitated the widespread sale of counterfeit products.

The tool developed here can contribute to future studies evaluating the possibility of detecting contaminated or falsified cosmetics products through monitoring via AMB techniques, DNA barcodes, etc. **[25–28].**

Research along these lines would also aid in gaining more insights into the trafficking of falsified cosmetics products. We would also be able to understand more clearly how not only consumers and the industry but also key stakeholders at the state level are impacted by such products. Meanwhile, this tool could also assist manufacturers in upholding their ethical responsibilities in terms of consumer safety when using cosmetics and facilitate interventions in the event that the use of these products causes harm.

In summary, based on the Lomé Initiative as well as other approaches, we present a useful and versatile tool for the detection of falsified cosmetic products **[23].** The instrument’s validity is strengthened by the use of multiple stages in its development, and we thus believe that the questionnaire can be of significant use in future research examining the prevalence of falsified cosmetics products, not only in the UAE but also globally. Our findings can in particular support community pharmacists in offering the right products to their customers, giving them peace of mind that these meet all the legal requirements, which is a crucial component of consumer safety. Our future work aims to engage with UAE pharmacy groups and the government, among others, to explore how we can use our findings to further support the monitoring of cosmetics products.

This study is subject to some limitations. As it was performed in a single country, this may have affected the generalizability to other contexts. Moreover, our tool has not yet been tested in practice. Nonetheless, the robust methodological approach employed here, we are confident that the tool will be effective both in the field and in other countries as falsified cosmetics products comprise a global issue.

## Conclusion

This study developed and validated a new scale to identify authentic and safe cosmetics and personal care products. The final instrument was a questionnaire using 24 items located in three dimensions (13 items on hazard information, eight items on product identity, and three items on handling and storage). The instrument is concise and easy to administer making it appropriate for use in general population research. Ultimately, this tool can facilitate enhanced collaboration between consumer health regulatory authorities and inspection authorities as well as improve consumer satisfaction and public participation.

## Supporting information

S1 File (ZIP)
