# Supplementary material for: Development and validation of a novel cosmetics safety assessment scale (CSAS): Factual understanding of cosmetic safety and fostering international awareness
Source: PLoS One. 2022 Nov 10;17(11):e0276938. doi: 10.1371/journal.pone.0276938 (PMC9648786; doi:10.1371/journal.pone.0276938)
Supplement: S1 File — (ZIP) [file pone.0276938.s001.zip › COSMETICS SAFETY ASSESSMENT SCALE (CSAS).docx]

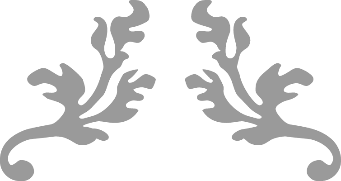


**Development and validation of a novel cosmetics safety assessment scale (CSAS): Factual understanding of cosmetic safety and fostering international awareness**


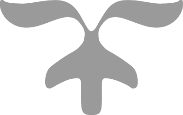


Development and validation of a novel cosmetics safety assessment scale (CSAS): Factual understanding of cosmetic safety and fostering international awareness

1. **Demographic characteristics**

| Demographic | Responses |
| --- | --- |
| Gender | Male  Female |
| Age group | 18 to 24 years  25 to 34 years  35 to 44 years  45 to 54 years  55 to 64 years  65 or older |
| Nationality | Emirati  Middle East  North Africa  South Asia  East Asia/Pacific  Central Asia  Europe  Africa  Western Europe  North America  Australia |
| Educational level | High school |
|  | Bachelor`s degree |

This tool is designed to help public including health professionals carry out a visual assessment of cosmetic for signs of unsafe, unregulated or falsified cosmetic product such as improper packaging, labelling or description of dosage. [**How frequently you do check or evaluate the following information on cosmetics’ label when you using/consuming cosmetic product? ].**

**How frequently you do check or evaluate the following information on cosmetics’ label when you buying cosmetic product?**

| **Hazards identification measure** | |
| --- | --- |
| 1. Cautionary statement “Extremely flammable aerosol” | Never Rarely Sometimes often Always |
| 1. Cautionary statement “Keep away from heat/sparks/open flames/hot surfaces” | Never Rarely Sometimes often Always |
| 1. Information concerning particular hazards for accidental ingestion | Never Rarely Sometimes often Always |
| 1. Information concerning particular hazards for accidental and prolonged inhalation | Never Rarely Sometimes often Always |
| 1. Warning “Keep out of reach of children” | Never Rarely Sometimes often Always |
| 1. Information concerning particular hazards for contact with skin/mucous membranes | Never Rarely Sometimes often Always |
| 1. Information concerning particular hazards for contact with eyes | Never Rarely Sometimes often Always |
| 1. Information on hazardous ingredients | Never Rarely Sometimes often Always |
| 1. Cautionary statement “Do not pierce or burn, even after use” | Never Rarely Sometimes often Always |
| 1. Information on conditions for safe storage | Never Rarely Sometimes often Always |
| 1. Restrictions on use on use by specific groups of consumers | Never Rarely Sometimes often Always |
| 1. List of ingredients or reference to enclosed or attached information about list of ingredients | Never Rarely Sometimes often Always |
| 1. Relevant identified used, recommended use, and restrictions on use | Never Rarely Sometimes often Always |
| **Cosmetic authentic measure** | |
| 1. Batch number | Never Rarely Sometimes often Always |
| 1. Barcode | Never Rarely Sometimes often Always |
| 1. Name and address of the manufacturer of the product | Never Rarely Sometimes often Always |
| 1. Registration of the product in the country by the concerned regulatory authority | Never Rarely Sometimes often Always |
| 1. Nominal content of the product | Never Rarely Sometimes often Always |
| 1. Country of origin | Never Rarely Sometimes often Always |
| 1. Cosmetic product claims and indications match with the active ingredients of the product | Never Rarely Sometimes often Always |
| 1. Premises and shops selling the cosmetics assure that the products meet the proper specifications throughout its shelf life | Never Rarely Sometimes often Always |
| **Handling measure** | |
| 1. Cosmetic container is safely sealed | Never Rarely Sometimes often Always |
| 1. Cosmetic container and closure protect the product from the outside environment | Never Rarely Sometimes often Always |
| 1. Container and the closure are appropriate for the cosmetic product inside | Never Rarely Sometimes often Always |

*Thank you*
